# Life-course pathways from childhood socioeconomic status to type 2 diabetes in mid-late Chinese adulthood

**DOI:** 10.1038/s41598-021-91768-1

**Published:** 2021-06-22

**Authors:** Xiaoning Zhang, Xue Jiang, Mengqi Sha, Qiong Zhou, Wen Li, Yuqing Guo, Zhengyan Ou, Junli Cao

**Affiliations:** 1 School of Management, Xuzhou Medical University, 209 Tongshan Road, Xuzhou, 221004 China; 2 School of Nursing, Capital Medical University, 10 YouAnMen Xitoutiao, Beijing, 100069 China; 3 Population, Policy and Practice Research and Teaching Department, UCL Great Ormond Street Institute of Child Health, 30 Guilford Street, London, WC1N 1EH UK; 4 Department of Pediatrics, The Affiliated Hospital of Zunyi Medical University, Zunyi, China; 5 School of International Education, Xuzhou Medical University, 209 Tongshan Road, Xuzhou, 221004 China; 6 Jiangsu Province Key Laboratory of Anesthesiology, Xuzhou Medical University, Xuzhou, 221004 Jiangsu China; 7 Jiangsu Province Key Laboratory of Anesthesia and Analgesia Application Technology, Xuzhou Medical University, Xuzhou, 221004 Jiangsu China

**Keywords:** Diseases, Health care

## Abstract

The relationship between childhood socioeconomic status (SES) and type 2 diabetes (T2D) remains inconclusive, and the pathways and mechanisms driving this relationship have yet to be clarified. This study aimed to examine the pathways linking childhood SES to T2D prevalence in mid-late adulthood in a low- and middle-income country. The incidence of T2D diagnosed in mid-late Chinese adulthood was assessed using self-reports from the Health and Retirement Longitudinal Study (CHARLS). Childhood SES was assessed by the education, occupation, survivorship of the parents and the financial situation of the family, whereas adulthood SES was assessed by education and wage. This study performed structural equation modeling to clarify the direct and indirect pathways from childhood SES to T2D via childhood health, childhood food shortage, adulthood SES and physical activity. A total of 15,132 participants were included, and the prevalence of T2D was 5.24%. This study found that childhood SES was directly associated with T2D in mid-late adulthood, the probability of developing T2D increased by 9.20% of the standard deviation for each decrease in standard deviation in childhood SES. Childhood SES was indirectly associated with T2D via adulthood SES, physical activity, childhood health and food shortage. Adulthood SES and physical activity mainly mediated the indirect pathway from childhood SES and T2D. This study showed direct and indirect pathways from disadvantaged childhood SES to increased risk of T2D in mid-late Chinese adulthood. Childhood SES, adulthood SES, physical activity, childhood health and food shortage were identified as life-course interventional targets that should be considered in the development of effective strategies to reduce the burden of T2D and SES-related health inequities in childhood.

## Introduction

Type 2 diabetes (T2D) has emerged as a major global public health concern, estimated to affect approximately 425 million (8.8%) adults worldwide, as reported by the International Diabetes Federation^1^. As one of the low- and middle-income countries (LMICs), China is the primary contributor to the global disease burden of T2D, the number of T2D patients has doubled in the last decade and reached 113.9 million in 2019^2^. Linked to enormous economic costs and human suffering^3^, T2D influences severe microvascular and macrovascular complications, as well as mental illnesses^4,5^, placing a heavy burden on healthcare systems globally^6^.

Adulthood socioeconomic status (SES) has been identified as a major determinant of T2D in both high-income countries (HICs) and LMICs^7^. Thus, the role of childhood SES in the development of late-life T2D has to be ascertained^8^. Childhood SES bears significance in increasing vulnerability as well as predicting SES and health trajectories across the life course^9^. A developmental perspective from early-life exposures is important to understand the association of childhood SES inequity with T2D^3^. Disadvantaged childhood SES may increase the proximal risk factors affecting health and distal risk factors influencing parenting capacities that subsequently constitute the risk factors affecting childhood disadvantages for the next generation^10^. The education, occupation, and survivorship of the parents, in addition to the financial situation of the family, are commonly used to measure childhood SES and are well validated in Chinese adults^11,12^. The survivorship of parents during childhood is associated with access to family SES and the emotional resources of children^13^. Paternal occupation and education indicate familial sociocultural background and SES resources^14,15^, which are associated with late-life diseases^12^, including adiposity^16^, dementia^17^, and T2D^18^; meanwhile, maternal education and occupation are positively related to the development and health of children^15,19^. Lower parental education and occupation were related to a higher risk of stroke among Germans^20^ and increased the odds of developing T2D among middle-aged Dutch adults^21^.

Evidence from England indicated that childhood SES was associated with a higher risk of T2D^8^; American adults with disadvantaged childhood SES were more likely to develop T2D^22^. Studies on the relationship between childhood SES and T2D are still insufficient and mostly originate from HICs; moreover, studies in LMICs receive limited attention^21^. Findings in HICs are not necessarily applicable to LMICs; small sample sizes, different sociocultural contexts with HICs, and inconsistent measures of childhood SES may limit generalization in LMICs^11^. Potential pathways from childhood SES to T2D in LMICs have yet to be ascertained. Further, previous studies often conducted traditional regression models to examine the relationship between childhood SES and T2D^8^ but rarely proposed pathways from childhood SES to T2D, particularly via childhood health, adulthood SES, and physical activity^23^. Structural equation modeling (SEM) is less frequently used to disentangle the direct and indirect pathways from childhood SES to T2D^3,21^, which is preferable to traditional statistical analyses. SEM exhibits the potential to simultaneously incorporate multiple variables and disentangle complex pathways, apart from identifying the mediating variables^24^.

Disadvantaged childhood SES can potentially contribute to poor childhood health by limiting access to necessary vaccinations, medications, and healthcare resources^25^. Poor childhood health largely contributes to late-life health outcomes^26^ and is associated with a 17% increase in the risk for T2D among older Mexicans^27^. Individuals with disadvantaged childhood SES have an increased likelihood of experiencing childhood food shortage^28^, which is linked to a range of late-life health outcomes^25^. Exposure to childhood food shortage, particularly prolonged hunger, is related to a 14.6% increase in the probability of being overweight and a 5.3% increase in the likelihood of being obese^29^, which is correlated with a higher risk for metabolic disorders, including T2D^29^.

Individuals with disadvantaged childhood SES may have limited access to education and consequently, well-paid jobs, thus maintaining their SES in adulthood^21^. Disadvantaged adulthood SES translated from disadvantaged childhood SES compromises access to convenient medical care, proper nourishment, educational opportunities, and psycho-social resources^10,30^, contributing to chronic stress and prompting the tendency toward developing late-life diseases^30,31^. Evidence from England revealed that adulthood SES mediated the relationship between childhood SES and adiposity^16^. Similar results were reported on the relationship between childhood SES and systemic inflammation^32^. Adulthood SES largely mediated the relationship between childhood SES and late-life mortality among Americans^33^.

Lifestyle behaviors are developed from childhood, when children are profoundly affected by the socioeconomic environment created by their parents^34^. Lifestyle behaviors, which mainly include smoking, drinking status and physical activity, have been associated with disadvantaged childhood SES^35^, which potentially exert cumulative effects on physiologic dysregulation and consequently, on the development of T2D^21,36^. Physical activity had a protective effect on decreasing the risk of T2D^37^. Smoking and limited physical activity could explain about a third of SES disparities in T2D prevalence in British^36^. Similarly, lifestyle behaviors mediated the relationship between childhood SES and heightened systemic inflammation in Swiss^32^.

The life-course approach has been widely used to explore the pathways of early-life disadvantages on late-life health outcomes^25^; however, this method has been rarely applied in T2D research^8^. The critical period model suggests that disadvantaged childhood SES is directly associated with T2D through the scarring effects on biological systems^30,39^. Disadvantaged childhood SES may increase the risk of chronic diseases from childhood through physiologic, psycho-social, behavioral differences, or combinations of these mechanisms^21^. The accumulative risk model highlights the effects of life-course cumulative risk factors, including disadvantaged exposure and SES during childhood, on determining health outcomes in late life^38^. Disadvantaged childhood SES could stimulate the development of unhealthy behaviors into adulthood, causing a cumulative increased risk for T2D^39^. The chains-of-risk model, also called the pathway model, hypothesizes that childhood SES may place individuals on divergent health trajectories by life-course exposures, including adulthood SES and lifestyle behaviors^25^. These life-course theoretical models are not mutually exclusive and can be used to evaluate the magnitude of pathways from childhood SES to T2D^8^.

Childhood SES has particular implications not only for understanding the current situation but also for inferring future health outcomes of patients in LMICs, who are more likely to be affected by their childhood experiences, compared with their counterparts in HICs^30^. China has undergone considerable SES changes for decades^40^, which may influence mid-late Chinese adulthood that is rendered vulnerable by disadvantaged childhood SES^25^. Chinese adults are characterized by disadvantaged living conditions, widespread social unrest, prolonged exposure to hunger, and infectious diseases in childhood that constitute health challenges^40^. Exposure to disadvantaged childhood SES may have shaped the life-course health trajectories of Chinese adults in different ways^25,41^. The following were observed in Chinese adults: disadvantaged childhood SES increased the risk of cognitive disorders^40^ and depression^26^; disadvantaged childhood health significantly predicted cognitive and physical impairment^42^; and childhood food shortage was associated with late-life glucose abnormalities^23^.

**Figure 1:**
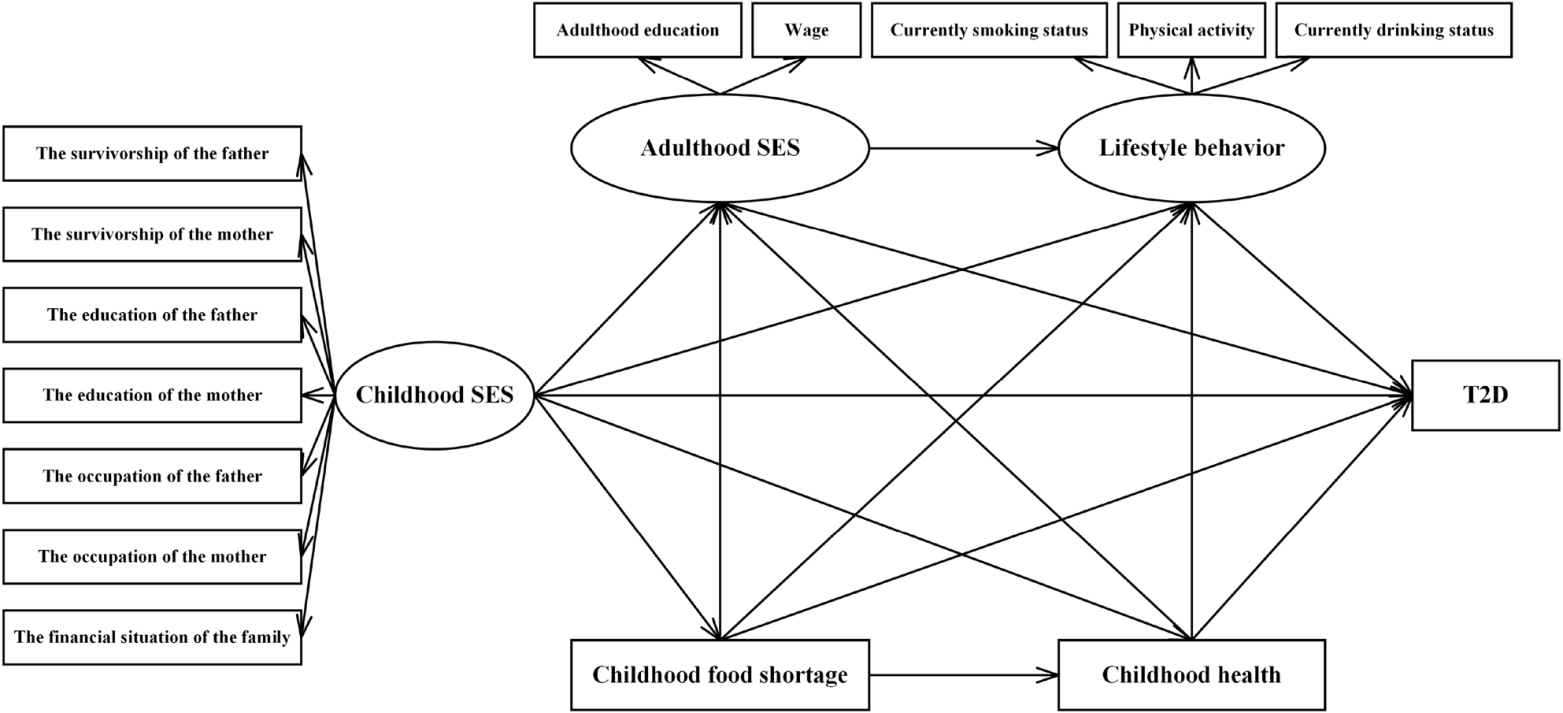
Hypothesized pathways from childhood SES to T2D in mid-late adulthood in the conceptual model. *SES* socioeconomic status, *T2D* type 2 diabetus.

Taken together, a comprehensive understanding of pathways linking childhood SES to T2D in mid-late Chinese adulthood is crucial to identify potential interventional targets, thereby reducing the risk for late-life T2D and health inequities from childhood^43^. The conceptual frameworks (Fig. 1) based on life-course theoretical models combined with a literature review were as follows: (1) a direct pathway may exist from childhood SES to T2D in mid-late Chinese adulthood; (2) Childhood food shortage and health may mediate the pathways from childhood SES to T2D; (3) Adulthood SES and lifestyle behaviors may mediate the indirect pathways from childhood SES to T2D.

## Methods

### Study design and participants

The data used in this study were from the 2014 and 2015 waves of the China Health and Retirement Longitudinal Study (CHARLS), a nationally representative survey among community-dwelling Chinese adults aged 45 and older^44^. CHARLS adopted a three-stage stratified probability-proportional-to-size sampling framework involving 10,803 households from 450 villages/residential communities in 28 provinces across mainland China^44^. CHARLS was approved by the Ethical Review Committee of Peking University and informed consent was obtained from each participant at the time of participation^44^. All methods were carried out in accordance with relevant guidelines and regulations. The 2014 wave was a life history survey of 20,543 participants and collected retrospective data on childhood SES (the education, occupation, survivorship of the parents and financial situation of the family), childhood health and food shortage^25^; the 2015 wave was a regular follow-up survey of 21,095 participants and collected data on adulthood SES (education and wage), lifestyle behaviors (drinking, smoking and physical activity), and health outcomes^28^. Participants in the 2014 and 2015 waves were combined based on IDs to trace their information on childhood SES, childhood food shortage, childhood health, adulthood SES, lifestyle behaviors, and T2D incidence. Participants aged 45 or younger or those with missing information on T2D and childhood SES were excluded. This study ultimately included 15,132 participants in the current analyses.

### Measurements

The incidence of T2D was assessed based on responses to the question: “Have you been diagnosed with T2D by a doctor?”, which had two options (“Yes” or “No”).

Seven variables were used to assess childhood SES: the financial situation of the family, education, occupation and survivorship of the father and the mother^12,25^. The financial situation of the family was assessed based on the question “When you were a child under 17 years old, how was the family’s financial situation?” and then categorized into “a lot better off than them”, “somewhat better off than them”,“same as them”, “somewhat worse off than them” and “a lot worse off than them”. This study classified the financial situation of the family as “not worse” (“a lot better off than them”,“somewhat better off than them” and “same as them”) and “worse” (“somewhat worse off than them” and “a lot worse off than them”). The survivorship of the father and the mother were dichotomized into “Yes” or “No” in response to the question “Was your father (mother) alive in your childhood?”. China being a largely agricultural society in the early twentieth century, the general education level was very low^19^, more than four-fifths (83.02%) of the mothers of the participants did not receive education, about 90% of the parents of the participants were farmers. Accordingly, the education of the mother and father were accordingly dichotomized into “received some education” or “no education,” and the occupation of the father and the mother were dichotomized into “non-agricultural” or “agricultural”^11,25^.

Childhood food shortage was assessed based on the question: “When you were a child before age 17 was there ever a time when your family did not have enough food to eat?” and dichotomized into “Yes” or “No”^26^.

Childhood health was self-reported based on the question: “How would you evaluate your health during childhood, up to and including age 15?” and categorized into “much healthier”, “somewhat healthier”, “fair”, “somewhat less healthy” and “much less healthy”. This study classified childhood health as “healthy” (“much healthier” and “somewhat healthier”), “fair” or “unhealthy” (“somewhat less healthy” and “much less healthy”)^26^.

Adulthood SES was assessed based on education and wage^14^. The education was grouped into four categories “illiterate”, “primary school”, “middle school” or “high school and above”. The wage was determined on the question “Have you received wage last year?” and dichotomized into “Yes” or “No”.

Lifestyle behaviors included smoking status, drinking status and physical activity. Currently drinking status was classified into “Yes” or “No” based on the question “Did you drink any alcoholic beverages in the past year?”; currently smoking status was classified into “Yes” or “No” based on the question “Did you have the habit of smoking cigarettes/smoking a pipe/chewing tobacco now?”. Physical activity was determined by the responses to the question “During a usual week, did you do any physical activities for at least 10 min continuously?” and dichotomized into “Yes” or “No”.

### Statistical analyses

Descriptive analyses and Spearman correlation analysis of variables were conducted using STATA 14.0, and the calculations and estimates of the SEM models were performed using MPlus7.4. No significant difference in the distribution of covariates was found between the final analysis sample and those with missing data in T2D and childhood SES. Frequencies (percentages) and mean values (standard deviation, SD) for the categorical and continuous variables were calculated. For descriptive statistics, Chi-squared tests and Fisher’s Exact tests were used to compare the categorical variables including gender, childhood SES, adulthood SES and lifestyle behaviors between participants with and without T2D. Spearman correlation analyses were performed to explore the correlation between childhood SES and T2D in mid-late adulthood. Based on the conceptual framework, this study developed the analytical model through the SEM approach to explore direct and indirect pathways between childhood SES and T2D. Confirmatory factor analysis was used to construct childhood SES and adulthood SES, and acceptable factor loadings were determined. Multiple modification indices were used to adjust and modify the SEM model to realize the best-fit model^45^. These indices included the comparative fit index (CFI), goodness-of-fit index (GFI), adjusted goodness-of-fit index (AGFI), standardized root mean square residual (SRMR), and the root–mean–square error of approximation (RMSEA). CFI ≥ 0.80, RMSEA and SRMR ≤ 0.06, GFI and AGFI > 0.90, indicating an acceptable model fitness^46^. Although χ^2^ values should be reported as one of the fit indices, they were highly sensitive to large sample sizes and thus were excluded^47^. The best-fitting SEM model included childhood health, childhood food shortage, adulthood SES including education and wage, and physical activity as mediating variables in indirect pathways from childhood SES to T2D. Bias-corrected bootstrapping (2000 bootstrap samples) was then used to test the statistical significance of the direct and indirect effects of each pathway in the model. The total effects were estimated using the sum of the direct and indirect effects, mathematically expressed as^48^: c = c′ + ab, where c = total effect, c′ = direct effect, ab = indirect effect. *P* < 0.05 indicated statistical significance.

### Ethical approval and consent to participate

CHARLS was approved by the Ethical Review Committee of Peking University and informed consent was obtained from each participant at the time of participation. All methods were carried out in accordance with relevant guidelines and regulations.

## Results

Table 1 presents the descriptive statistics and comparison by T2D for the analyzed variables. T2D incidence was reported by 15,132 participants (7011 males and 8121 females), with the average age of 60.56 ± 10.15 years. A total of 793 participants were diagnosed with T2D by the doctor (5.24%). For the childhood SES variables, 2533 (16.75%) and 4224 (27.91%) participants reported the survivorship of their fathers and mothers during childhood, 7974 (52.52%) and 12,562 (83.02%) participants reported their fathers and mothers did not receive education, 13,890 (91.79%) and 12,622 (83.41%) participants reported the occupation of their fathers and mothers were farmers, and 6120 participants reported the financial situation of the family as worse. Of 10,207 participants (67.45%) experienced childhood food shortage, 13,073 (86.39%) reported childhood health status as poor/fair. Individuals whose father/mother did not receive education were more likely to develop T2D than those whose father/mother received some education (*p* < 0.05). Individuals who reported the survivorship of father/mother during childhood were less likely to develop T2D than those who reported death of father/mother (*p* < 0.05). Individuals who experienced childhood food shortage were more likely to develop T2D than those who did not experience food shortage (*p* < 0.05). Individuals with lower education and did not receive wage were more likely to develop T2D than those with higher education and received wage (*p* < 0.01). Individuals who did not do physical activity were more likely to develop T2D than those did physical activity, and female were more likely to develop T2D than males (*p* < 0.05).

**Table 1:** Descriptive statistics of the participants (*N* = 15,132).

Variables	Overall	T2D^a^		χ^2^	*p* value
		No	Yes		
**Gender**		14,339 (94.76)	793 (5.24)	9.18	0.002
Male	7011 (46.33)	6685 (95.35)	326 (4.65)		
Female	8121 (53.67)	7654 (94.25)	467 (5.75)		
**Childhood SES**^**b**^					
**The survivorship of the father**				50.64	< 0.001
No	12,597 (83.25)	11,864 (94.18)	733 (5.82)		
Yes	2535 (16.75)	2475 (97.63)	60 (2.37)		
**The survivorship of the mother**				49.32	< 0.001
No	10,908 (72.09)	10,250 (93.97)	658 (6.03)		
Yes	4224 (27.91)	4089 (96.80)	135 (3.20)		
**The education of the father**				7.52	0.006
No education	7947 (52.52)	7493 (94.29)	454 (5.71)		
Received some education	7185 (47.48)	6846 (95.28)	339 (4.72)		
**The education of the mother**				5.75	0.016
No education	12,562 (83.02)	11,879 (94.56)	683 (5.44)		
Received some education	2570 (16.98)	2460 (95.72)	110 (4.28)		
**The occupation of the father**				0.15	0.699
Non-agricultural	1242 (8.21)	1174 (94.52)	68 (5.48)		
Agricultural	13,890 (91.79)	13,165 (94.78)	725 (5.22)		
**The occupation of the mother**				2.93	0.087
Non-agricultural	2510 (16.59)	2361 (94.06)	149 (5.94)		
Agricultural	12,622 (83.41)	11,978 (94.90)	644 (5.10)		
**The financial situation of the family**				0.89	0.344
Worse	6120 (40.44)	5812 (94.97)	308 (5.03)		
Not worse	9012 (59.56)	8527 (94.62)	485 (5.38)		
**Childhood food shortage**				5.13	0.023
Yes	10,207 (67.45)	9643 (94.47)	564 (5.53)		
No	4925 (32.55)	4696 (95.35)	229 (4.65)		
**Childhood health**				1.13	0.570
Healthy	2059 (13.61)	116 (5.63)	1943 (94.37)		
Fair	7777 (51.39)	395 (5.08)	7382 (94.92)		
Unhealthy	5296 (35.00)	282 (5.32)	5014 (94.68)		
**Adulthood SES**					
**Education**				51.53	< 0.001
Illiterate	5789 (38.26)	5465 (94.40)	324 (5.60)		
Primary school	5103 (33.72)	4794 (93.94)	309 (6.06)		
Middle school	1138 (7.52)	1063 (93.41)	75 (6.59)		
Higher school and above	3102 (20.50)	3017 (97.26)	85 (2.74)		
**Wage**				16.33	< 0.001
No	12,133 (80.18)	11,453 (94.40)	680 (5.60)		
Yes	2999 (19.82)	2886 (96.23)	113 (3.77)		
**Physical activity**				19.94	< 0.001
No	12,402 (81.96)	11,705 (94.38)	697 (5.62)		
Yes	2730 (18.04)	2634 (96.48)	96 (3.52)		
**Currently smoking status**				0.25	0.616
Yes	5408 (35.74)	5118 (94.64)	290 (5.36)		
No	9724 (64.26)	9221 (94.83)	503 (5.17)		
**Currently drinking status**				41.19	< 0.001
Yes	5201 (34.37)	5012 (96.37)	189 (3.63)		
No	9931 (65.63)	9327 (93.92)	604 (6.08)		

^a^Type 2 diabetes; ^b^Socioeconomic status.

Table 2 presents the correlations between variables of childhood SES, childhood food shortage, childhood health, adulthood SES, physical activity and T2D. T2D was positively correlated with the survivorship of the father (*r* = 0.058, *p* < 0.05) and mother (*r* = 0.057, *p* < 0.05), receiving no education of the father (*r* = 0.022, *p* < 0.05) and mother (*r* = 0.019, *p* < 0.05). Childhood food shortage was positively correlated with higher risk for T2D (*r* = 0.018, *p* < 0.05).

**Table 2:** Correlation matrix of the variables used in this study.

	(1)	(2)	(3)	(4)	(5)	(6)	(7)	(8)	(9)	(10)	(11)	(12)	(13)	(14)	(15)
The survivorship of the father (1)	1.000														
The survivorship of the mother (2)	0.382**	1.000													
The education of the mother (3)	0.173**	0.162**	1.000												
The education of the father (4)	0.157**	0.131**	0.337**	1.000											
The occupation of the father (5)	0.099**	0.080**	0.005	0.014	1.000										
The occupation of the mother (6)	0.065**	0.095**	− 0.040**	− 0.013	0.166**	1.000									
The financial situation of the family (7)	0.069**	0.054**	0.067**	0.092**	0.106**	0.007	1.000								
Childhood food shortage (8)	0.128**	0.125**	0.117**	0.086**	0.036**	− 0.039**	0.208**	1.000							
Childhood health (9)	0.005	− 0.004	0.013	0.005	0.042**	≺0.001	0.100**	0.051**	1.000						
Adulthood education (10)	0.214**	0.231**	0.190**	0.167**	0.077**	0.004	0.084**	0.144**	0.049**	1.000					
Wage (11)	0.120**	0.157**	0.102**	0.088**	0.048**	0.022**	0.035**	0.074**	0.037**	0.198**	1.000				
Physical activity (12)	0.036**	0.042**	0.007	− 0.023**	0.014	0.047**	− 0.040**	− 0.033**	0.001	0.009	0.054**	1.000			
Currently drinking status (13)	0.009	0.039**	0.028**	− 0.018*	0.027**	− 0.010	0.006	0.021**	0.021**	− 0.142**	− 0.154**	− 0.072**	1.000		
Currently smoking status (14)	0.122**	0.125**	0.064**	0.067**	0.005	0.010	0.029**	0.107**	− 0.016*	0.046**	− 0.037**	− 0.035**	0.290**	1.000	
T2D^a^ (15)	0.058**	0.057**	0.019*	0.022**	0.003	0.014	− 0.008	0.018*	0.001	0.034**	0.033**	0.036**	− 0.052**	0.004	1.000

^a^Type 2 diabetes.*p < 0.05; **p < 0.01.

### SEM results

**Figure 2:**
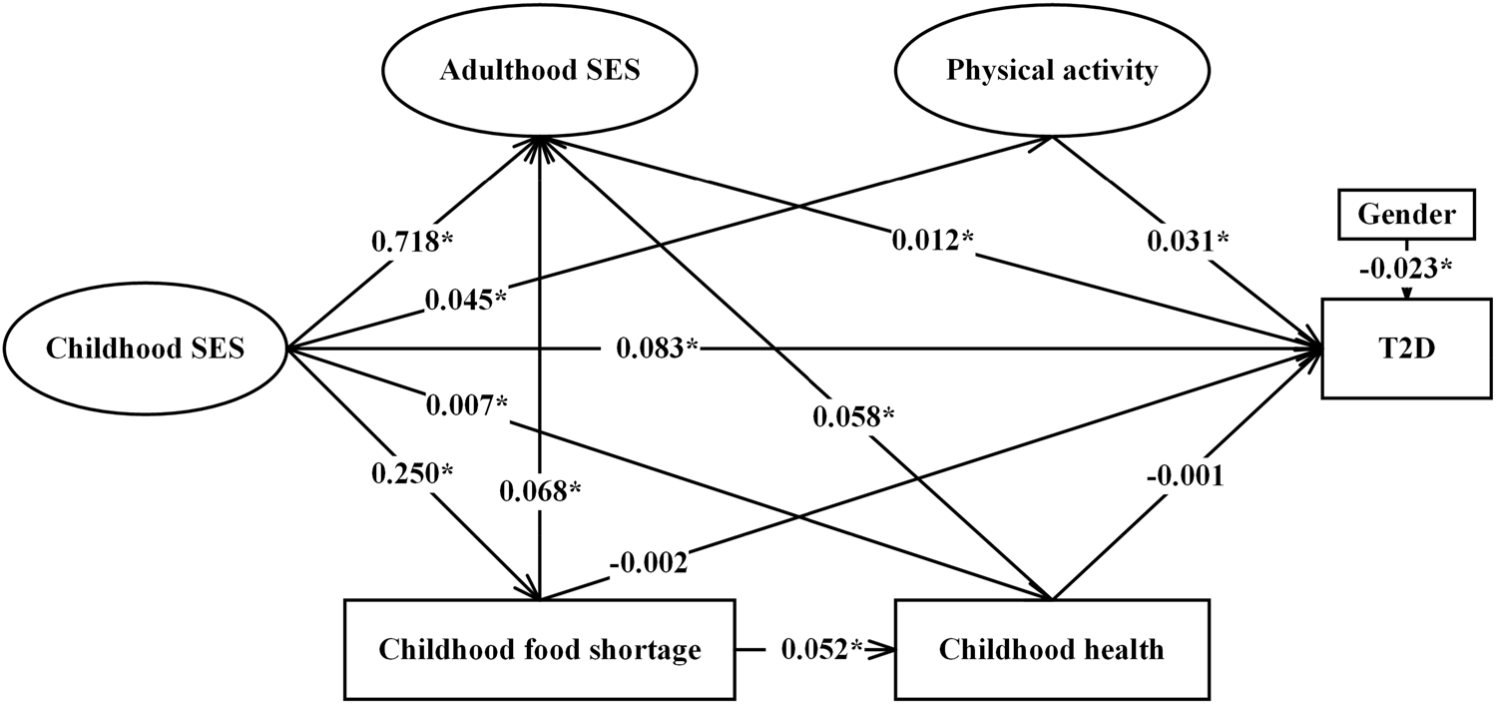
Standardized estimates of the pathways from childhood SES to T2D in mid-late adulthood in the model. *p < 0.05. *SES* socioeconomic status, *T2D* type 2 diabetus. Fitting of the model: CFI=0.809; AGFI = 0.970; GFI = 0.978; RMSEA = 0.054; SRMR = 0.040.

Figure 2 presents the standardized path estimates of the pathways from childhood SES to late-life T2D, Tables 3 and 4 present the standardized estimates of the direct, indirect, and total effects of childhood SES on late-life T2D prevalence as well as the specific effects through multiple pathways of childhood health, childhood food shortage, adulthood SES and physical activity. Childhood SES exerted significant direct (*β* = 0.083, *p* < 0.05), indirect (*β* = 0.009, *p* < 0.05) and total (*β* = 0.092, *p* < 0.05) effects on T2D; 90.21% of the total effect of childhood SES on T2D was direct, 9.78% was indirect and mainly mediated through adulthood SES and physical activity. Specifically, disadvantaged childhood SES positively predicted T2D via disadvantaged adulthood SES with an estimated indirect effect of 0.009 (*p* < 0.05). Disadvantaged childhood SES positively predicted T2D via limited physical activity, with an estimated indirect effect of 0.002 (*p* < 0.05). Disadvantaged childhood SES significantly predicted T2D via the sequential mediating variables of childhood food shortage and adulthood SES or via the sequential mediating variables of childhood health and adulthood SES. Disadvantaged childhood SES significantly predicted T2D via the sequential mediating variables of childhood food shortage, childhood health and adulthood SES. Physical activity had the greatest total effects on T2D (*β* = 0.031, *p* < 0.05), followed by adulthood SES (*β* = 0.012, *p* < 0.05). The female gender was significantly associated with a higher risk for T2D (*β* = − 0.023, *p* < 0.05). The goodness-of-fit indices of the best-fitting model were acceptable with the following values: GFI, 0.978; AGFI, 0.970; CFI, 0.809; SRMR, 0.040 and RMSEA, 0.054.

**Table 3:** Standardized indirect path estimates from childhood SES to late-life T2D of the model.

Pathways	Standardized path estimates
Childhood SES^a^ → adulthood SES → T2D^b^	0.009*
Childhood SES → poor childhood health → T2D	− 0.001
Childhood SES → childhood food shortage → T2D	− 0.001
Childhood SES → physical activity → T2D	0.002*
Childhood SES → childhood food shortage → adulthood SES → T2D	≺0.001*
Childhood SES → childhood food shortage → poor childhood health → T2D	≺0.001*
Childhood SES → poor childhood health → adulthood SES → T2D	≺0.001*
Childhood SES → childhood food shortage → poor childhood health → adulthood SES → T2D	≺0.001*

^a^Socioeconomic status; ^b^Type 2 diabetes. *p < 0.05.

**Table 4:** Standardized direct effect, indirect and total effect of the variables on late-life T2D^a^ prevalence.

Variables	Standardized direct effects	Standardized indirect effects	Standardized total effects
Childhood SES^b^	0.083*	0.009*	0.092*
Poor childhood health	− 0.001	0.001*	≺0.001*
Childhood food shortage	− 0.002	0.001*	− 0.001*
Adulthood SES	0.012*	–	0.012*
Physical activity	0.031*	–	0.031*
Gender	− 0.023*	–	− 0.023*

^a^Type 2 diabetes; ^b^Socioeconomic status. *p < 0.05.

## Discussion

To the best of our knowledge, this study is the first to examine the potential pathways from childhood SES to T2D by using a nationally representative sample and the SEM approach in mid-late Chinese adulthood, verifying the existence of life-course theoretical models in an LMIC. This study showed significant direct and indirect pathways from childhood SES to T2D. Disadvantaged childhood SES was indirectly associated with T2D via adulthood SES, physical activity, childhood health and childhood food shortage, which were identified as valuable life-course intervention targets to reduce the risk for T2D. Both direct and indirect pathways from childhood SES to T2D may provide important arguments for policymakers to improve childhood SES, consequently reducing health inequities in early life.

Consistent with the conceptual framework, this study confirmed that disadvantaged childhood SES was directly associated with a higher risk for T2D in mid-late Chinese adulthood, the finding supported the evidence from HICs^21^. A Finnish population-based study showed that individuals with disadvantaged childhood SES were more likely to develop late-life T2D^49^; a similar association was reported in Americans^50^. Consistent evidence from Latin America, the Caribbean, and the United States revealed that disadvantaged childhood SES increased the risk for late-life T2D^18,51^. The direct effect of childhood SES on T2D indicated that childhood was a critical period across the life course and supported the critical period model. Disadvantaged childhood SES activated and modified inflammation processes and hypothalamic–pituitary–adrenal regulations^52^, which may facilitate the development of T2D^50^. Disadvantaged childhood SES limited the opportunities for obtaining sufficient physical, psychosocial, and medical resources and increased the risk for low-quality parental interactions and adverse stimulations^53^. These adverse experiences constitute adverse interpersonal experiences and social exclusion^26^, which may be contributory to the risk for T2D^54^.

In accordance with the previous study^3^, the current study showed that childhood SES was indirectly associated with T2D in mid-late Chinese adulthood via adulthood SES, supporting the chains-of-risk and accumulative risk models. The finding highlighted the importance of T2D-related SES pathways and indicated that advantaged adulthood SES could compensate for the consequences of disadvantaged childhood SES in the risk for T2D^21^. Individuals with disadvantaged childhood SES, which is relatively stable across the life course, tend to acquire limited access to high-quality education, obtain few job opportunities to positions requiring higher education, and experience poor living conditions, their disadvantaged SES may be retained in adulthood^55^. The level of education and wage in adulthood played important mediating effects in the relationship between childhood SES and late-life health^15^. Disadvantaged adulthood SES was closely related to poor health literacy, which is associated with poor late-life health outcomes, and individuals with inadequate literacy were less likely to acquire preventive information on T2D^56^. Individuals with disadvantaged adulthood SES could have a higher risk for social isolation, less social support, and increased stress, which are associated with a higher risk for glucometabolic impairment, prompting the development of T2D^56^.

The current study clarified the indirect pathway from disadvantaged childhood SES to T2D through limited physical activity, supporting the chains-of-risk and accumulative risk models^3^, which agreed with previous studies involving middle-aged Americans^57^ and older Englishmen^8^. Inadequate studies have been reported on the pathway from childhood SES to T2D mediated by physical activity in adulthood. Meanwhile, the present study provided novel findings regarding this association and indicated that the differences in lifestyle behaviors, particularly physical activity, had existed in childhood and persisted into adulthood^35^. The underlying reasons for the mediating role of physical activity in the pathway from childhood SES to T2D are complex^35^. Disadvantaged childhood SES is associated with poor health consciousness, negative health beliefs, and suboptimal lifestyle behaviors, which may affect internal control and access to health-related information on T2D^21^. Existing cultural norms and the provision of healthy products for individuals with disadvantaged SES are associated with the acquisition of exercise facilities^58^, which may influence the adoption of healthy behaviors, particularly physical activity, in childhood and the development of T2D^57^. Individuals with disadvantaged childhood SES have inferior knowledge and skills necessary to develop healthy lifestyle behaviors, they may learn and accept fewer intervention programs for T2D prevention^58^.

The current study showed the sequential mediating effects of childhood food shortage and childhood health in indirect pathways from childhood SES to T2D. Experiencing food shortage and having poor health during childhood were associated with poor cognitive performance, delayed school attendance, dropping out of school, decreased schooling completion, and subsequent lower wages in adulthood^59^. Childhood health may drain socioeconomic resources preventing investment in healthcare and limiting the capacity to acquire skills necessary to shape adulthood SES^60^, which is associated with an increased risk for T2D^56^. The mild effects of childhood health and food shortage mediated the indirect pathways between childhood SES and the risk for T2D. Improving these aspects, which may influence health, education, and economic benefits in adulthood, may not preclude the achievement of progress^59^. The present study also found that females were more likely to develop late-life T2D, which was consistent with an Indian study reporting a higher prevalence of T2D in females than in males^61^.

### Interventions from the life course perspective

The pathways from childhood SES to T2D indicated that early-life intervention is crucial to human development and promotes avoidance and resilience against T2D development^10^. Exposure to early-life disadvantages is irreversible and weakly supported by an emphasis on investment in childhood^62^. Interventions to promote resilience in children with disadvantaged SES contribute to the development of optimal mental and physical health, equipping children better and earlier to endure, adapt to, and thrive after exposure to childhood disadvantages. Increasing focus on the multidimensional interventional approach, which encompasses education, healthcare, food availability, and physical activity, to prevent T2D are highly needed^43^. Current substantial investment in education and improvement in the school system could be leveraged to formulate optimal childhood health interventions^62^. Policymakers in LMICs should improve childhood health and food availability as high priorities in T2D prevention, education, and SES agenda to alleviate the negative effects of disadvantaged childhood SES^59^. The school system in LMICs with limited healthcare coverage may provide a cost-efficient platform for health promotion and the delivery of healthcare resources and services for children with disadvantaged SES^63^. School-based interventions should combine teachings in health education both inside and outside classrooms, including health curricula incorporating families and communities; these supplements, which may facilitate the whole life-course health and reduce the risk for T2D^62,63^. School-based programs, such as meals, vaccination, and annual deworming programs, should be conducted to promote school participation, improve childhood health and food availability, which is a cost-effective intervention for females to provide intergenerational benefits^62^. Consequently, far-reaching effects are exhibited on reducing the risk for T2D^13^. Evidence suggests that substantial returns on investment in education and employment^62^ and multi-sectorial strategies should be targeted at providing educational opportunities and vocational training during adulthood^11^. Expanding access to higher education and offering more employment opportunities for females may reap substantial benefits, including the provision of comprehensive health knowledge and the development of optimal lifestyle behaviors^62^, as well as narrow the SES disparities in behaviors^35^. Community-, school- and family-based health-promoting interventions for lifestyle behaviors (such as physical activity), targeted at children with disadvantaged SES, may prevent the risk of T2D^36^.

### Strengths and limitations

This study has several strengths. To the best of our knowledge, this is the first study to examine the direct and indirect pathways from childhood SES to T2D in a LIMC, using the largest nationally representative sample in mid-late Chinese adulthood. Moreover, this study innovatively used the SEM approach to disentangle the direct and indirect pathways from childhood SES to T2D, and identified the mediating effects of disadvantaged childhood health, food shortage, disadvantaged adulthood SES and limited physical activity. SEM addresses methodological challenges in estimating direct and indirect effects via multiple pathways, and is valuable in examining multiple complex intercorrelations, identifying mediating variables and understanding pathways to propose targeted interventions in the critical life period^46^.

The findings in this study should be interpreted cautiously for several limitations. First, this cross-sectional study is limited to determining the definitive causal relationship between childhood SES and T2D. Although the retrospective information was reported as reasonably reliable^64^, measurements of childhood conditions were based on retrospective self-reports with ineluctable recall bias. Second, this study was limited by the available variables and responses coded in the existing CHARLS database, such as the categorization of occupation and education of parents, smoking status and drinking status. Potential variables, such as dietary habits, history of smoking, and drinking status that are associated with T2D might not be included in the database, limiting the conclusion of pathways from childhood SES to T2D. This study attempted to adjust biomarkers, including the body mass index, blood pressure and heart rate, drinking and smoking status in the SEM model; however, the results showed a poor modeling fit and insignificant associations. Finally, individuals suffering from extremely disadvantaged childhood SES and childhood health might not have reached 45 years old, underestimating the relationship and bias in the pathways between childhood SES and T2D^26^.

## Conclusion

This study is thus far the first study to clarify the direct and indirect pathways from childhood SES to T2D in mid-late Chinese adulthood. Childhood health and food shortage, adulthood SES, and physical activity mediated the indirect pathways. The findings in this study showed that disadvantaged childhood SES may propel individuals on unhealthy trajectories, such as T2D, and suggested that attention be particularly directed toward preventing T2D and thereby reduce health disparities on individuals with disadvantaged childhood SES. This study identified adulthood SES, physical activity, childhood health and food shortage, which could be targeted interventions to reduce the risk of T2D in mid-late adulthood caused by disadvantaged childhood SES. Future birth-cohort studies with a national representative sample in LMICs are recommended to explore the underlying pathways between childhood SES and late-life health outcomes, as well as to design and modify targeted interventions to reduce health inequities in childhood.

## Data Availability

The data that support the findings of this study are available from the China Health and Retirement Longitudinal Study (CHARLS), but restrictions apply to the availability of these data, which were used under license for the current study. Data are however available from the authors upon reasonable request and with permission of CHARLS research team. The authors had no special access privileges in accessing data from CHARLS.

## Abbreviations

T2D: Type 2 diabetes
SES: Socioeconomic status
SEM: Structural equation modeling
CHARLS: China Health and Retirement Longitudinal Study
LMICs: Low- and middle-income countries
HICs: High-income countries
CFI: Comparative fit index
SRMR: Standardized root mean square residual
GFI: Goodness-of-fit index
AGFI: Adjusted goodness-of-fit index
RMSEA: Root mean square error of approximation

## References

[1] Saeedi P (2019). Global and regional diabetes prevalence estimates for 2019 and projections for 2030 and 2045: Results from the International Diabetes Federation Diabetes Atlas, 9(th) edition. Diabetes Res. Clin. Pract..

[2] Ma RCW (2018). Epidemiology of diabetes and diabetic complications in China. Diabetologia.

[3] Tsenkova V, Pudrovska T, Karlamangla A (2014). Childhood socioeconomic disadvantage and prediabetes and diabetes in later life: A study of biopsychosocial pathways. Psychosom. Med..

[4] Stringhini S (2013). Association of lifecourse socioeconomic status with chronic inflammation and type 2 diabetes risk: The Whitehall II prospective cohort study. PLoS Med..

[5] Haffner SM, Lehto S, Rönnemaa T, Pyörälä K, Laakso M (1998). Mortality from coronary heart disease in subjects with type 2 diabetes and in nondiabetic subjects with and without prior myocardial infarction. N. Engl. J. Med..

[6] Huang X, Wang L, Yue R, Ding N, Yang H (2020). Large dosage Huangqin (Scutellaria) and Huanglian (Rhizoma Coptidis) for T2DM: A protocol of systematic review and meta-analysis of randomized clinical trials. Medicine (Baltimore).

[7] Wemrell M, Bennet L, Merlo J (2019). Understanding the complexity of socioeconomic disparities in type 2 diabetes risk: A study of 43 million people in Sweden. BMJ Open Diabetes Res. Care.

[8] Pikhartova J, Blane D, Netuveli G (2014). The role of childhood social position in adult type 2 diabetes: Evidence from the English Longitudinal Study of Ageing. BMC Public Health.

[9] Gauffin K, Hemmingsson T, Hjern A (2013). The effect of childhood socioeconomic position on alcohol-related disorders later in life: A Swedish national cohort study. J. Epidemiol. Community Health.

[10] Nurius PS, Fleming CM, Brindle E (2017). Life course pathways from adverse childhood experiences to adult physical health: A structural equation model. J. Aging Health.

[11] Zhong Y, Wang J, Nicholas S (2017). Gender, childhood and adult socioeconomic inequalities in functional disability among Chinese older adults. Int. J. Equity Health.

[12] Ming W, Gu D (2011). The effects of childhood, adult, and community socioeconomic conditions on health and mortality among older adults in China. Demography.

[13] Yi Z, Gu D, Land K (2007). The association of childhood socioeconomic conditions with healthy longevity at the oldest-old ages in China. Demography.

[14] Shen K, Zeng Y (2014). Direct and indirect effects of childhood conditions on survival and health among male and female elderly in China. Soc. Sci. Med..

[15] Galobardes B, Smith GD, Lynch JW (2006). Systematic review of the influence of childhood socioeconomic circumstances on risk for cardiovascular disease in adulthood. Ann. Epidemiol..

[16] Strand B, Murray E, Guralnik J, Hardy R, Kuh D (2010). Childhood social class and adult adiposity and blood-pressure trajectories 36–53 years: Gender-specific results from a British birth cohort. J. Epidemiol. Community Health.

[17] Wang XJ (2019). Early-life risk factors for dementia and cognitive impairment in later life: A systematic review and meta-analysis. J. Alzheimers Dis..

[18] Beckles GL (2019). Life course socioeconomic position, allostatic load, and incidence of type 2 diabetes among African American Adults: The Jackson Heart Study, 2000–04 to 2012. Ethn. Dis..

[19] Zhang Z, Liu J, Li L, Xu H (2018). The long arm of childhood in China: Early-life conditions and cognitive function among middle-aged and older adults. J. Aging Health.

[20] Becher H (2016). Socioeconomic conditions in childhood, adolescence, and adulthood and the risk of ischemic stroke. Stroke.

[21] Derks IPM (2017). The association of early life socioeconomic conditions with prediabetes and type 2 diabetes: Results from the Maastricht study. Int. J. Equity Health.

[22] Maty SC, Lynch JW, Raghunathan TE, Kaplan GA (2008). Childhood socioeconomic position, gender, adult body mass index, and incidence of type 2 diabetes mellitus over 34 years in the Alameda County Study. Am. J. Public Health.

[23] Zhang Y (2018). Risk of hyperglycemia and diabetes after early-life famine exposure: A cross-sectional survey in northeastern China. Int. J. Environ Res. Public Health.

[24] Shah R, Goldstein SM (2006). Use of structural equation modeling in operations management research: Looking back and forward. J. Oper. Manag..

[25] Peele ME (2019). Domains of childhood disadvantage and functional limitation trajectories among midlife men and women in China. J. Aging Health.

[26] Tian F, Meng SS, Qiu P (2019). Childhood adversities and mid-late depressive symptoms over the life course: Evidence from the China health and retirement longitudinal study. J. Affect. Disord..

[27] Kohler IV, Soldo BJ (2005). Childhood predictors of late-life diabetes: The case of Mexico. Soc. Biol..

[28] Zhang X, Chen S (2019). Association of childhood socioeconomic status with edentulism among Chinese in mid-late adulthood. BMC Oral Health.

[29] McEniry M, Samper-Ternent R, Flórez CE, Cano-Gutierrez C (2019). Early life displacement due to armed conflict and violence, early nutrition, and older adult hypertension, diabetes, and obesity in the middle-income country of Colombia. J. Aging Health.

[30] McEniry M (2012). Early-life conditions and older adult health in low- and middle-income countries: A review. J. Dev. Orig. Health Dis..

[31] Insaf TZ, Strogatz DS, Yucel RM, Chasantaber L, Shaw BA (2014). Associations between race, lifecourse socioeconomic position and prevalence of diabetes among US women and men: results from a population-based panel study. J. Epidemiol. Community Health.

[32] Carmeli C (2020). Mechanisms of life-course socioeconomic inequalities in adult systemic inflammation: Findings from two cohort studies. Soc. Sci. Med..

[33] Hayward MD, Gorman BK (2004). The long arm of childhood: The influence of early-life social conditions on men's mortality. Demography.

[34] Marttila-Tornio K, Mannikko N, Ruotsalainen H, Miettunen J, Kaariainen M (2020). Lower parental socioeconomic status in childhood and adolescence predicts unhealthy health behaviour patterns in adolescence in Northern Finland. Scand. J. Caring Sci..

[35] Puolakka E (2018). Childhood socioeconomic status and lifetime health behaviors: The Young Finns Study. Int. J. Cardiol..

[36] Stringhini S (2012). Contribution of modifiable risk factors to social inequalities in type 2 diabetes: Prospective Whitehall II cohort study. BMJ.

[37] McEniry M (2011). Infant mortality, season of birth and the health of older Puerto Rican adults. Soc. Sci. Med..

[38] Yoav BS, Diana K (2002). A life course approach to chronic disease epidemiology: Conceptual models, empirical challenges and interdisciplinary perspectives. Int. J. Epidemiol..

[39] Joseph JJ (2017). Modifiable lifestyle risk factors and incident diabetes in African Americans. Am. J. Prev. Med..

[40] Yang L, Wang Z (2020). Early-life conditions and cognitive function in middle-and old-aged Chinese adults: A longitudinal study. Int. J. Environ. Res. Public Health.

[41] McEniry M, McDermott J (2015). Early-Life conditions, rapid demographic changes, and older adult health in the developing world. Biodemography Soc. Biol..

[42] Wang Q, Zhang H, Rizzo JA, Fang H (2018). The effect of childhood health status on adult health in China. Int. J. Environ. Res. Public Health.

[43] Huang H (2015). Adverse childhood experiences and risk of type 2 diabetes: A systematic review and meta-analysis. Metabolism.

[44] Zhao Y, Hu Y, Smith JP, Strauss J, Yang G (2012). Cohort profile: The China Health and Retirement Longitudinal Study (CHARLS). Int. J. Epidemiol..

[45] Browne M, Cudeck R (1992). Alternative ways of assessing model fit. Sociol. Methods Res..

[46] Kline, R. B. (2005). *Methodology in the social sciences. Principles and practice of structural equation modeling (2nd ed)*. Guilford Press. https://psycnet.apa.org/record/2005-03476-000https://journals.sagepub.com/doi/10.1177/1049731509336986.

[47] Schermelleh-Engel K, Moosbrugger H, Müller H (2003). Evaluating the fit of structural equation models: Tests of significance and descriptive goodness-of-fit measures. Methods Psychol. Res. Online.

[48] Vanderweele TJ (2015). Mediation analysis: A practitioner's guide. Annu. Rev. Public Health.

[49] Puolakka E, Pahkala K, Laitinen TT, Magnussen CG, Juonala M (2016). Childhood socioeconomic status in predicting metabolic syndrome and glucose abnormalities in adulthood: The cardiovascular risk in Young Finns Study. Diabetes Care.

[50] Nandi A, Glymour MM, Kawachi I, VanderWeele TJ (2012). Using marginal structural models to estimate the direct effect of adverse childhood social conditions on onset of heart disease, diabetes, and stroke. Epidemiology.

[51] Palloni A, McEniry M, Wong R, Peláez M (2006). The tide to come: Elderly health in Latin America and the Caribbean. J. Aging Health.

[52] Meaney M, Szyf M, Seckl J (2007). Epigenetic mechanisms of perinatal programming of hypothalamic-pituitary-adrenal function and health. Trends Mol. Med..

[53] Cohen S, Janicki-Deverts D, Chen E, Matthews KA (2010). Childhood socioeconomic status and adult health. Ann. N. Y. Acad. Sci..

[54] Raphael D (2011). Poverty in childhood and adverse health outcomes in adulthood. Maturitas.

[55] Pavela G (2017). Is childhood socioeconomic status independently associated with adult BMI after accounting for adult and neighborhood socioeconomic status?. PLoS ONE.

[56] Brown AF, Ettner SL, Piette J, Weinberger M, Beckles GL (2004). Socioeconomic position and health among persons with diabetes mellitus: A conceptual framework and review of the literature. Epidemiol. Rev..

[57] Tsenkova VK, Lee C, Boylan JM (2017). Childhood socioeconomic disadvantage, occupational, leisure-time, and household physical activity, and diabetes in adulthood. J. Phys. Act. Health.

[58] Emmons KM, Barbeau EM, Gutheil C, Stryker JE, Stoddard AM (2006). Social influences, social context, and health behaviors among working-class, multi-ethnic adults. Health Educ. Behav..

[59] Victora CG (2008). Maternal and child undernutrition: Consequences for adult health and human capital. Lancet (London, England).

[60] Warren JR, Knies L, Haas S, Hernandez EM (2012). The impact of childhood sickness on adult socioeconomic outcomes: Evidence from late 19th century America. Soc. Sci. Med..

[61] Scavini M (2003). Prevalence of diabetes is higher among female than male Zuni indians. Diabetes Care.

[62] Bundy DAP (2018). Investment in child and adolescent health and development: Key messages from Disease Control Priorities, 3rd Edition. Lancet.

[63] Shackleton N (2016). School-based interventions going beyond health education to promote adolescent health: Systematic review of reviews. J. Adolesc. Health.

[64] Luo Y, Waite LJ (2005). The impact of childhood and adult SES on physical, mental, and cognitive well-being in later life. J. Gerontol. Ser. B Psychol. Sci. Soc. Sci..

